# A mediating model of mindfulness, sense of purpose in life and mental health among Chinese graduate students

**DOI:** 10.1186/s40359-022-00799-4

**Published:** 2022-04-06

**Authors:** Yibin Wang, Tian Tian, Junjie Wang

**Affiliations:** 1 School of Foreign Language, Jiangsu Second Normal University, Nanjing, 211200 China; 2 Educational Sciences College, Shaanxi University of Technology, No. 1 East First Ring Road, MO, Hanzhong City, 723000 Shaanxi Province China

**Keywords:** Mindfulness, Sense of purpose in life, Mental health, Graduate student

## Abstract

The purpose of the present study is to examine the relationship between mindfulness and mental health of graduate students and the mediating effects of sense of purpose in life on mindfulness and mental health. The participants include 419 graduate students from 6 universities in China, and there are 190 males and 229 females. The Hayes Process is adopted to analyze the effects of the sense of purpose in life on mindfulness and mental health of graduate students. The results reveal that mindfulness can effect the mental health of graduate students positively and significantly. The sense of purpose in life is found to mediate the relationship between mindfulness and mental health. In further moderated mediation analyses, the effect of mindfulness on mental health can be adjusted by family economic condition. The type of degree can adjust the effect of mindfulness on sense of purpose, and academic Interest can adjust the mediating effect of sense of purpose. Finally, this study discusses several empirical and methodological implications of the findings.

## Introduction

### Mental health of graduate students

Graduate students’ mental heath conditions have severer and severer, because of the combined impact of the continuous expansion of the graduate students’ enrollment and multiple pressures. Taking graduate students as the research subjects, this study explore the positive psychological factors that affect the mental health of graduate students and explore the interaction mechanism of mindfulness and mental health, which has unique theoretical and practical significance [1]. Graduate students are a core group for the development of a country [2]. Therefore, universities not only need to continue optimizing their education model, but also pay attention to the students’ mental health and other non-intellectual factors. Obviously, according to ‘The Dual-Factor Model of Mental Health’, the mental health of an individual is mainly characterized by positive factor indicators and negative factor indicators [3, 4]. In previous studies, Symptom-cheklist-90 (SCL-90) was often used to measure the mental health of graduate students. In the past five years, there were 2645 related studies, 1779 master's theses and 84 doctoral dissertations in the Chinese journal website, using "college students" and "SCL 90" as the subject terms. However, these survey results covered only the negative part of the psychology of graduate students, such as anxiety and depression, and positive factors were generally ignored [5]. In most existing researches, more attention has been drawn to “What factors can affect the mental health of graduate students?” The research outcomes have proved that stress, psychological problems and family situation are the major influential factor [6]. The compensating model is usually used to explain people’s mental health state, representing the compensatory activity of positive characteristics toward negative factors [6]. According to the empirical research, a variety of positive traits can be used to inhibit the generation of negative factors. For instance, perceived social support can inhibit the production of negative psychology in graduate students [7, 8]. The “problem-based” research concept causes circumscribed and rapid educational practices, which has been demonstrated in previous studies [9]. For graduate students in China, the economic situation of each family and the academic Interests of each graduate student play important roles in the demographic aspects that influence the mental health of the graduate students [10–12]. Therefore, in this study, the positive mental health model of graduate students was selected as the research variable for analysis. This model has three dimensions: positive emotional experience, good psychological quality and adequate social support [12, 13].

### Relationship between mindfulness and mental health of graduate students

Mindfulness, the cognitive way of an individual to keep open to new things, one of the first introductions of mindfulness into health psychology was in the 1970s psychologists Ellen Lange et al. [14]. Compared with mindlessness, mindfulness is a mode of positive and hardworking conscious thinking and a flexible mental state. Another way of saying, mindfulness consists of a purposeful and receptive focus on the present, it’s dynamic [15]. Bishop et al. view positive thinking as a state-like trait that includes both "Self-regulation to Attention" and "Orientation to One’s Experience" [16, 17]. In cross-cultural studies, Chinese researchers primarily discuss "which experiences can be perceived, and be adopted a specific orientation or attitude" [18]. Therefore, the study understands the mindfulness in terms of social cognition. It is explained that “What states when you are aware of your inner activity”, and includes finding new meaning, constructing a meaning and being open to things. It also refers to the need for the experience to be characterized by curiosity, openness and acceptance. Studies have shown that graduate students have high self-expectation and motivation for achievement and are faced with pressures from the academic background, academic development, employment, and interpersonal difficulties at the same time in China [19, 20]. These pressures usually lead to negative emotions in graduate students. Consequently, the number of graduate students with mental health problems is dramatically increasing year after year.

Many studies have demonstrated a positive relationship between mindfulness and mental health in different age groups, including children, adolescents, youth and adults [21–24]. Shapiro et al. proposed the mental model of positive thinking as a mechanism of positive perception for health [25]. Through mindfulness, individuals improve their self-adjustment abilities so that they acquire positive emotions and positive psychological qualities [26, 27]. And it has been shown that mindfulness may improve cognitive functioning to obtain positive experience of academic achievements [28]. Further research has shown that mindfulness can enhance active detection, expand the range of awareness, and increase the proportion of positive emotions perceived [29, 30], which directly improve peoples’ mental health [31, 32]. As a result, peoples' stress is released, negative emotions are regulated, and well-being is increased. In addition, research has found that the perception of being expanded due to mindfulness can effectively retain social support [33]. The training of mindfulness can help students adapt and promote personal mental health and academic performance. People with higher levels of mindfulness have a clearer understanding of themselves and their current environment [34–36], and it is easier to identify the nature of the problem and the solution in a more rational and clearer manner.

### The mediating role of the sense of purpose in life

The purpose of life is a general intention that has individual subjective will. It is meaningful to ego and affects the external environment [37]. Usually, the purpose of life is understood as a high-dimensional psychological structure integrated and constructed to satisfy needs and desires and pursue happiness [38]. In addition, the purpose of life can help us understand the origin and reality of human health as well as the success and happiness in career development [39]. Therefore, it can be seen clearly that both theoretical research and empirical research have gradually clarified the key role of the purpose of life in the active development of people [40]. “The sense of purpose in life” measures whether an individual’s life goal is stable and sustainable and whether it is rooted in the active exploration of internal and external self in environmental events and social learning [38]. As a cognitive mode, mindfulness plays an important role in the exploration of purpose in life. It is the main reason why this study takes the sense of purpose in life as an intermediary variable. In the Self-determination Theory [41], the internal dynamic model was used to explain self-behavior, which was tendentiously selected after self-cognition and environmental information were fully integrated [42]. Mindfulness is a behavioral process in which new information is accepted through multiple perspectives and categories are reconstructed by multi-perspective approaches. It is an indispensable characteristic in the process of self-decision. Individual behavior is driven through a broader goal called “the purpose in life”. It is the development intention to point and transcend ego, having the same positive impact on the construction of graduate students' mental health [43]. On the one hand, mindfulness promotes the efficient operation of the internal drive to explore the value of life by ascent of cognition. When the purpose in life is molded, the efficiency of self-exploration is increased to seek information from environment [44, 45]. At the same time, the threshold of awareness is opened up to increase the sensitivity that can clarify the purpose in life. On the other hand, a clear purpose in life has a positive effect on the positive factors of students’ mental health. A clear purpose can help acquire positive emotional experience, cultivate positive psychology and accumulate social support when graduate students with a distinct purpose in life have positively predicted life events in order to experience abundant positive emotions [46, 47] (Özkan Çıkrıkçı, 2020). Meanwhile, positive psychological qualities can be cultivated for individuals by the sense of purpose in life, such as subjective well-being, psychological adaptation, and self-regulation, which promotes the cultivation of individuals in the pursuit of self-improvement. In addition, adolescents’ peer interaction and parenting styles are directly influenced by their purposes in life, and they generally obtain different degrees of social support [48]. It has been confirmed that the purpose in life has a positive effect on the perceived social support [49].

**Fig. 1:**
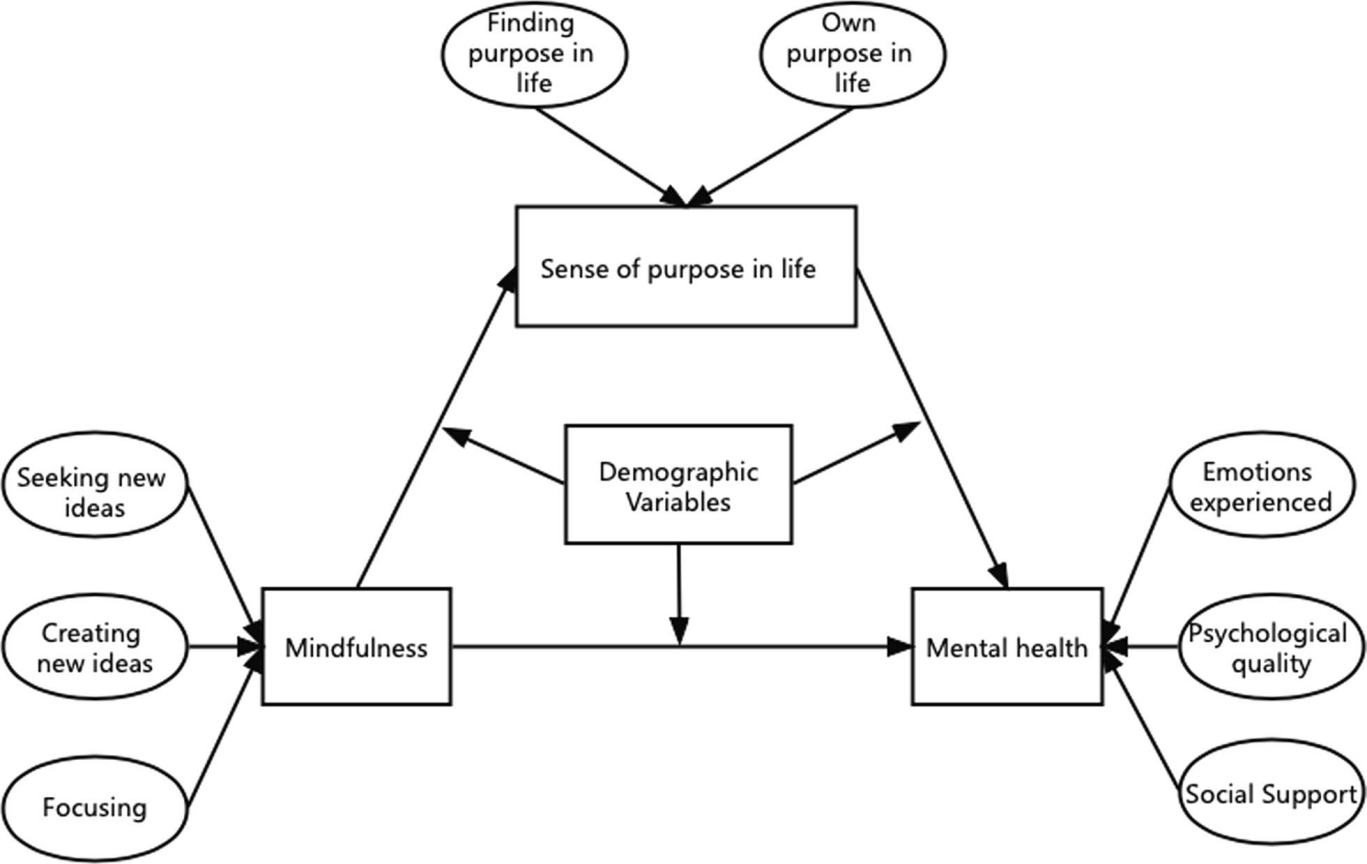
The model of this study

Therefore, this study examined the relationship between mindfulness and the mental health of graduate students, with a focus on whether they had any intermediary relationship. Based on the Positive-Factor Model of Mental Health, the following hypotheses were proposed: (1) mindfulness could have an effect on the mental health of graduate students through the sense of purpose in life. (2) Family economic situation and academic Interests have a great impact on the model. The theoretical model is shown in Fig. 1.

## Method

### Participants

In this study, an online questionnaire survey platform called “Questionnaire Star”, as well as offline questionnaires, were used to collect data of graduate students from Shaanxi University of Technology, Shaanxi University of Science and Technology, Lanzhou University of Technology, Xiangtan University, Nanjing Normal University, and Sun Yat-sen University (based on Chinese university rankings). Due to the COVID-19 pandemic and the restriction of close social contact, offline data collection was aborted.

After unqualified questionnaires (e.g., questionnaires that were filled out randomly and unfinished) were filtered out, a total of 419 valid samples were collected, including 229 from professional students (training mode is mainly practical) and 190 from academic students(academic research is focused on training). According to “the 2019 China Statistical Yearbook” and “the Criteria for Identifying Students in Poverty in Higher Education”, among the 419 subjects, 80 had poor family financial status (recognized as a poor student by your school) and 30 had good family financial status (annual household income above RMB 150,000), 309 came from middle-income families (not recognized as a needy student and with an annual family income of less than RMB 150,000). According to the questionnaire feedbacks from the 419 graduate students, 314 were interested in their majors and the rest were not. This study was approved by the ethics committee of Jiangsu Second normal University. The participants were informed about the purpose of the study and, prior to the start of the study, were assured that their privacy would be protected. An anonymous self-administered questionnaire was completed by students voluntarily. Written informed consent was obtained from all participants. All methods in the current study were carried out in accordance with relevant guidelines and regulations.

### Measures

#### Graduate Students Mental Health Scale

In the research, the "Graduate Students Mental Health Scale" was used [19]. The research was conducted from three dimensions: positive emotional experience, positive psychological quality, and good social support, and the response scale ranged from 1 (strongly disagree) to 5 (strongly agree). The instrument consisted of 47 items. In the present sample, the total Cronbach α coefficient of the scale was 0.978, and the internal consistent reliability of each sub-scale was respectively 0.936, 0.961 and 0.955, which showed high reliability.

#### Langer Mindfulness Scale (LMS)

In this study, the Chinese version of the Langer Mindfulness Scale (LMS) developed by Ellen Langer et, al and translated and revised by Hu Jing et al. in 2014 was used [50, 51]. It was tested to be applicable to measure the perception of mindfulness [52]. It can be used to explain novelty seeking, novelty producing, and engagement from the aspect of social-cognition. The LMS scale consisted of 13 items, including the three factors of seeking new ideas, creating new ideas and focusing. The response scale ranged from 1 (strongly disagree) to 7 (strongly agree). LMS displayed high internal consistency (Cronbach’s = 0.792). The score for each item was summed to generate the total scores of mindfulness.

#### Life Purpose Scale

The "Life Purpose Scale" applied in the study was compiled by Lan Gongrui et, al, and was continuously revised during use [40]. It measured the two dimensions of finding a sense of purpose in life and having a sense of purpose in life through 18 items, among which 5 items were reversed calculation. The response scale ranged from 1 (very inappropriate) to 7 (very appropriate), and a higher score represented a stronger sense of purpose in life. In the present sample, the Cronbach α coefficient of the total scale and two sub-scales were 0.811, 0.563, and 0.727 respectively.

### Procedure

Data were collected from November 2020 to January 2021. Graduate students were required to complete a series of questionnaires regarding mindfulness, mental health and sense of purpose in life. All subjects were informed that their responses would be kept confidential and would in no way affect their academic evaluation.

SPSS20.0 was used to analyze the data for exploratory analysis, descriptive analysis, variation test, correlation analysis and regression analysis. The process program (Hayes) was used, and the Bootstrap method was used for the significance test.

## Results

### Common method biases test

We examined common method biases by Harman’ s single-factor analysis, finding that there were 11 items with characteristic roots greater than 1, with a total contribution rate of 42.26%; 22.96% was the variation contribution rate of the first factor. The results suggested that there was no severe common method bias in this study.

### Difference tests

A t-test showed that there was a significant gender difference in Mindfulness (*t* = 2.97, *p* = 0.03) and that male participants (M = 4.72, SD = 0.66) were higher than were female participants (M = 4.54, SD = 0.56). Moreover, there was a significant grade difference in mental heath (*F* = 3.665, *p* = 0.027); the participants in grade 1 (M = 3.80, SD = 0.45) were more heath than those in grade 2 and 3 (M = 3.70, SD = 0.46).

### Mesomeric effect test

Table 1 shows the descriptive statistics and the correlation coefficients between variables. Mindfulness and sense of purpose in life were both positively correlated with mental health. A hypothesis testing was carried out on the basis of the correlation of various variables, which was based on the test method of intermediary effect proposed by Wen Zhonglin and Ye Baojuan (2014). The mediating role of the sense of purpose in life between mindfulness and the mental health of graduate students was tested, and the total effect of mindfulness on the mental health of graduate students was also examined. As indicated by the results in Table 2, the hypothetical intermediary model did not contain "0" in the 95% confidence interval. Mindfulness positively predicted the sense of purpose in life. The sense of purpose in life as well as mindfulness also positively predicted the mental health of graduate students. The sense of purpose in life had a significant mediating effect between mindfulness and the mental health of graduate students. The direct effect of specializing in the mental health of graduate students was 0.0374, the indirect effect was 0.0268 × 0.0561 = 0.0015; the total effect was 0.0366; and the ratio of the indirect effect to the total effect was 0.041.

**Table 1:** Means, standard deviations, and correlations for the variables

Variable	M	SD	Mindfulness	Sense of purpose in life	Mental health
Mindfulness	4.62	0.62	1		
Sense of purpose in life	3.54	0.41	0.573**	1	
Mental health	4.03	0.56	0.572**	0.696**	1

*p< 0.05, **p< 0.01 and ***p<0.001

**Table 2:** The mediating and regulating of the model

Variable	Estimate	Lower	Upper	*P*
Mindfulness → sense of purpose in life	0.0268	0.3299	0.4351	0.000***
Sense of purpose in life → mental health	0.0561	0.6397	0.8602	0.000***
Mindfulness → mental health	0.0374	0.1608	0.3080	0.000***
Family economic condition × mindfulness → mental health	0.0688	− 0.2956	− 0.0250	0.0204*
Type of degree × mindfulness → sense of purpose in life	0.0537	− 0.2601	− 0.0491	0.004**
Academic interest × sense of purpose in life → mental health	0.1605	0.3624	0.9933	0.000***

*p< 0.05, **p< 0.01 and ***p<0.001

Further, the differences of demographic variables in each path of the model were tested. The results showed that they were significantly different in family economic status, type of academic degrees, and academic Interests. Mindfulness had a weaker influence on the mental health of the graduate students with better family economic situation. The mindfulness of academic graduates had a greater impact on their sense of purpose in life than that of professional graduates. In addition, the sense of purpose in life had less influence on the mental health of the graduate students with a higher academic Interest.

## Discussion

### Analysis of variance in demographic variables

In this study, male participants' mindfulnes were more than female participants. Past study argued that mindfulness shows more openness to new things, new ideas, etc., and is more prominent in men; male graduate students are better able to show exploration and innovation, so they are more likely to choose engineering majors [51]. Meanwhile, the mental health of first-year graduate students was significantly higher than that of senior graduate students, and consistent with previous findings [8, 12, 53]. As the length of study increases, there is a significant increase in academic pressure on graduate students, which can affect their mental health; there are also adverse emotions arising from employment difficulties, which have been shown to be influential factors in the mental health of graduate students [9, 54, 55].

### Mediating roles of the sense of purpose in life

As is hypothesized, this study demonstrated that mindfulness had a direct impact on the mental health of graduate students, and the sense of purpose in life had a partial mediating effect on concentrating and the mental health of graduate students. Mindfulness influenced the mental health of graduate students not only directly but also indirectly through the sense of purpose in life. The intermediary model was explained from the following perspectives. On the one hand, from the perspective of social cognition, mindfulness is the characteristics of individuals with a high degree of participation and sensitivity to recognize, reconstruct, innovate and recognize in social situations. It is a mode of thinking formed by continuous adjusting under the influence of cognition. The components of open thinking and acceptance of new things in its ego structure provide cognition and ability for individuals in the process of pursuing life goals. Individual’s intentions can be extensively excavated through an open mind and thought transformation to perceive the social environment and then stimulate the internal motivation to achieve personal values and gradually influence the external world in the social interaction of realizing their intentions. The whole process is added to more powerful positive factors in the formation of the purpose in life, so that it becomes more comprehensive and efficient. On the other hand, according to the dual-factor model of mental health, happiness is an important indicator of individual mental health, and the purpose of life is the optimal key factor in the study of happiness. The result of this study showed that the sense of purpose in life can strongly predict mental health of the graduate students. In this study, the effect size of the sense of purpose in life on the mental health of graduate students was 0.0561, which generally explained the phenomenon. A clear sense of purpose in life can enable graduate students to actively acquire positive factors from the social interaction environment and accumulate rich positive experiences and social support, thereby driving them to achieve better self-development and maintain a higher level of mental health.

### Regulating effect of demographic variables

The study found that some demographic factors had a moderating effect on the research model. The effect of mindfulness on the mental health of graduate students can be regulated significantly by family economic conditions, and the effect of mindfulness on mental health was the smallest and insignificant for graduate students from rich families. This moderating effect showed that the family economic status affected the effect of mindfulness on the mental health of graduate students. Mindfulness could adjust cognization of people to abtain positive psychological experience, which is connection with student's early experience. It is derived from family environment and educational background [55] (Ludwig DS, Kabat-Zinn J, 2008). Graduate students with rich family economic conditions accumulate diversified experience to deal with possible incidents, than the others could be a challage whose family is not rich. In the dual-factor model of mental health, the main factor representing negative experience was depression, and family economic status was an indicator of the survival of family functions. Studies have shown that the interaction between mindfulness and family conditions can significantly inhibit individual depression and alleviate the damage caused by insufficient family function. The interaction also promoted the acquisition of positive emotional experiences. In the model of this study, graduate students from poor families needed more active awareness and self-adjustment to obtain positive emotional experience and good social support, whereas graduate students from rich families got more social support and a wider range of sources of positive emotional experiences. Consequently, it was not obvious to promote mental health by mindfulness. In addition, the effect that mindfulness had on the sense of purpose in life can be adjusted significantly by the type of academic degrees, due to the large gap between the academic system and training mode of academic graduate students and professional graduate student. Academic graduate students focus more on the deep system learning of subject theory, while professional graduate students focus more on the application of system theory. With the differences of the two types of degrees compared, the cultivation of academic master's degree was more value-oriented and required graduate students to create new things from many perspectives. At the same time, more students chose to become academic graduate students, who were recommended and extremely interested in professional subjects or have plans for their own professional development. Their sense of purpose in life was more stable than that of professional graduate students. They had a wider range of development and they judged their own purpose in life more decisively. Finally, the effect of the sense of purpose in life on the mental health of graduate students can be adjusted by the interest in the selected major. The role of graduate students’ sense of purpose in life and mental health was mainly realized through happiness brought by the sense of purpose in life and the positive experience gained in the process of actively seeking self-worth and social study. Therefore, students who chose their majors based on their academic Interests had more initiative and focus in their academic development, which strengthened the sense of purpose in life and continued to give graduate students motivation and incentive to make higher levels of achievement [40].

## Conclusion

The following conclusions were drawn from this study.

1. Mindfulness can seem to positively and significantly affect the mental health of graduate students; (2) The sense of purpose in life partially mediates the relationship between the idea of mindfulness and the mental health of graduate students; (3) The family economic situation can moderate the role of mindfulness on the mental health of graduate students. The type of master's degree can moderate the path of mindfulness on the sense of purpose in life, and the academic Interest can moderate the path of the sense of purpose in life on the mental health of graduate students. It certainly deserves further study. Diverse and more participants will make a greater contribution to this area, with active controls and using objective measures. Future research will discuss whether mindfulness training can be an effective intervention to the mental health of graduate students.

## Data Availability

The raw datasets during the current study are not publicly available due the parent database to which this study is subordinate is not publicly available at this time but the data result are available from the corresponding author on reasonable request.
